# The association between biomarkers and clinical outcomes in novel coronavirus pneumonia in a US cohort

**DOI:** 10.2217/bmm-2020-0309

**Published:** 2020-07-17

**Authors:** Shant Ayanian, Juan Reyes, Lei Lynn, Karolyn Teufel

**Affiliations:** ^1^The George Washington University School of Medicine & Health Sciences, Division of Hospital Medicine, The GW Medical Faculty Associates, DC 20037, USA

**Keywords:** biomarkers, COVID-19, CRP, D-dimer, ferritin, infectious diseases, interleukin-6, LDH

## Abstract

**Aim:** To describe the association between D-dimer, CRP, IL-6, ferritin, LDH and the clinical outcomes in a cohort of 299 novel coronavirus disease (COVID-19) patients treated on the inpatient medical service at a university hospital in the District of Columbia (DC, USA). **Methodology/results:** In this retrospective study, we included all laboratory confirmed COVID-19 adults admitted to the inpatient medicine service at the George Washington University Hospital between March 12, 2020 and May 9, 2020. We analyzed the association of biomarkers on intensive care unit transfer, intubation and mortality. Threshold values for all biomarkers were found to be statistically significant and independently associated with higher odds of clinical deterioration and death. **Conclusion:** Laboratory markers of inflammation and coagulopathy can help clinicians identify patients who are at high risk for clinical deterioration in COVID-19.

A viral pneumonia of an unknown cause was detected in the city of Wuhan, China and first reported to the WHO Country Office in China on 31 December 2019 [1]. In the USA, the virus was first seen in January 2020, in a traveler who returned from China [2]. By February 2020, WHO had given the virus a name: the novel coronavirus 2019, shorthand COVID-19. In early March 2020, shortly after the first cluster of cases was diagnosed in the Western USA [3], WHO declared a pandemic.

New York City was the first major metropolitan area on the US East coast to see a large number of COVID-19 patients. By early March, the virus was confirmed in the District of Columbia [4]. Shortly thereafter, the first cases were diagnosed at George Washington University Hospital (GWUH) in Washington, DC. In the following 2 months, 299 cases of confirmed COVID-19 were admitted to our hospital.

Previous studies of COVID-19 have identified various biologic markers associated with poor prognosis. An association between mortality as well as elevated inflammatory markers and coagulation functional indices were first reported in a retrospective cohort study of 138 patients with COVID-19 in Wuhan, China [5]. In this study, higher D-dimer and IL-6 levels were observed in patients requiring ICU admission, in patients with acute respiratory distress syndrome, and in nonsurvivors. Also published in March 2020, a second retrospective analysis of 191 patients found that elevated levels of LDH, ferritin, D-dimer and IL-6 were associated with mortality [6]. This group reported an 18-fold increase in the odds of in-hospital death in patients admitted with a D-dimer greater than 1 μg/l. In a subsequent retrospective analysis conducted at Tongji Hospital, Tang *et al.* demonstrated that of 183 COVID-19 patients, ≥70% of the nonsurvivors had an elevated D-dimer on admission [7]. In a commentary on Tang's data, Canadian Dr. Lillicrap concluded, *“The observations of Tang and colleagues provide early evidence that enhanced vigilance is required to identify the emergence of disseminated intravascular coagulopathy (DIC) in novel Coronavirus (2019‐nCoV) pneumonia patients”* [8].

Based on these and other reports in the literature [9], clinicians in the US have been measuring a variety of biologic markers in COVID-19 patients. The clinical impact of these biological markers on outcomes in the US population remains unclear. At GWUH, during the first few weeks of the COVID-19 pandemic, hospitals typically measured markers of inflammation and coagulopathy, but no standardized protocol for laboratory testing had been established. Our team sought to examine the association between these markers and clinical outcomes of our COVID-19 patients, with the goal of identifying which markers had clinical utility in assessing prognosis.

## Methods

All 299 adult (≥18 years of age) patients diagnosed with COVID-19 were admitted to the medicinal service at GWUH between March 12, 2020 and May 9, 2020 and were included in this retrospective study. These were the only two inclusion criteria used for this study. The COVID-19 diagnosis was based on one of the three platforms available for severe acute respiratory syndrome coronavirus 2 (SARS-CoV2) diagnostic testing at GWUH and when applicable, the test used by the department of health of the District of Columbia. All tests used were approved by the federal US FDA. All patients admitted to GWUH were placed in a special COVID-19 unit.

The Electronic Medical Records of the hospital were used to extract and record relevant clinical and laboratory data using a prespecified case report form. Patient age, sex, BMI, comorbidities and medications were recorded. Laboratory tests were performed either in the emergency room prior to admission or after the admission orders, and patients were placed in one of the special COVID-19 units. In addition to routine admission laboratory studies, CRP, D-dimer, IL-6, ferritin and LDH were recorded. Further, the maximum oxygen requirements prior to transfer to ICU, transfer to the ICU, necessity for mechanical ventilation and discharge status were noted. At the time of our study, no reliable risk estimator for COVID-19 severity of illness had been developed. Patients illness severity was assessed by the admitting physician and subsequently followed clinically.

## Statistical analysis

The data analysis was conducted using statistical software R. Given the sample size and the need for reliable markers for disease severity, the continuous variables were transformed into categorical variables. For IL-6, ferritin, CRP and LDH, the median of each group was used as the threshold (IL-6 ≥60 pg/ml [normal range 0.0–15.5 pg/ml], ferritin ≥450 ng/ml [normal range 20–450 ng/ml] and CRP ≥ 90 mg/l [normal range 0.0-9.0–mg/l], and LDH is 1200 units/l [normal 400–800 units/l]). For D-dimer (normal range 0.20–0.28 μg/ml), a threshold level of 3 μg/ml was used. This level had been reported in the recent literature from China as having a higher specificity for predicting thrombi [10].

After the key characteristics of the variables were studied, a logistic regression model was fitted with D-dimer, IL-6, LDH, ferritin and CRP with ICU admission, intubation and death sequentially. As all the coefficients of these markers were found to be statistically significant, the odds ratio of all three outcomes were calculated for patients with the categorical variables of IL-6, D-dimer, ferritin, LDH and CRP.

A stratification analysis was carried out with every one of the comorbid conditions recorded. These conditions were classified by the Elixhauser disease severity index and grouped into eight categories of interest. A weighted Agency for Healthcare Research and Quality (AHRQ) disease severity index was also calculated and later transformed into a binary variable with 1 indicating patients with a score higher than 10. Furthermore, a stratification with regard to sex and BMI > 30 was carried out. The mean age of patients within each outcome category was also compared.

## Results

A total of 299 patients with confirmed COVID-19 positive results were admitted to the hospital service at GWUH between March 12th and May 9th, 2020. Of these patients, 200 had all the biomarkers of interest measured by our clinicians. The median follow-up period was 8 days. 69 patients (23%) were transferred to the ICU, 39 patients (13%) required intubation and 71 patients (23.7%) died. The key laboratory findings for the five biomarkers can be found in Table 1, and the comorbid conditions are illustrated in Table 2. The markers were measured a few times during hospitalization and the peak value was used during analysis.

**Table 1.** Biomarkers studied with key characteristics.

Variable studied	Median	Standard deviation	Number of patients with available results
IL-6 (normal range 0.0–15.5 pg/ml)^†^	65 pg/ml	446.6 pg/ml	222
D-dimer (normal range 0.20–0.28 μg/ml)	2.1 μg/ml	4.7 μg/ml	248
Ferritin (normal range 20–450 ng/ml)	446 ng/ml	1822 ng/ml	252
CRP (normal range 0.0–9.0 mg/l)	90 mg/l	132.9 mg/l	241
LDH (normal range 600–800 units/l)	974 units/l	954.2 units/l	254

^†^ IL-6 performed at LabCorp, NC, USA. All other labs performed at the George Washington University Hospital.

**Table 2.** Comorbidities and outcomes, odds ratios.

Characteristics	Prevalence	ICU admission	Intubation	Death
Sex = M	54%	2.6 (1.4, 4.6)^†^	2.4 (1.1, 5.0)^†^	1.4 (1.0, 2.2)
BMI > 30	29%	1.2 (0.7, 2.4)	1.3 (0.6, 2.9)	0.6 (0.3, 1.4)
Hypertension	68%	2.4 (1.2, 4.5)^†^	2.8 (1.1, 7.0)^†^	2.8 (1.4, 5.5)^†^
Diabetes	46%	1.3 (0.8, 2.2)	1.7 (0.8, 3.2)	1.2 (0.7, 2.1)
Cardiac disease	46%	1.9 (1.8, 3.3)^†^	2.0 (1.0, 4.2)^†^	3.6 (2.0, 6.3)^†^
CVA	17%	1.9 (1.3, 3.0)^†^	3.2 (1.6, 6.4)^†^	4.5(2.5, 8.0)^†^
COPD	29%	1.2 (0.7, 2.2)	1.3 (0.7, 2.8)	1.7 (1, 3)
CKD	29%	1.5 (0.9, 2.7)	0.9 (0.4, 1.9)	2.3 (1.4, 4.0)^†^
HIV	4%	NA	NA	0.27 (0, 2)
Cancer	6%	1.6 (0.5, 4.6)	0.9 (0.1, 3.3)	1.5 (0.5, 4.4)

^†^ indicates statistical significance at a p-value of 0.05.

COPD: Chronic obstructive pulmonary disease; CKD: Chronic kidney disease; CVA: Cerebrovascular disease; HIV: Human immunodeficiency virus; ICU: Intensive care unit; M: Male.

**Figure 1.**
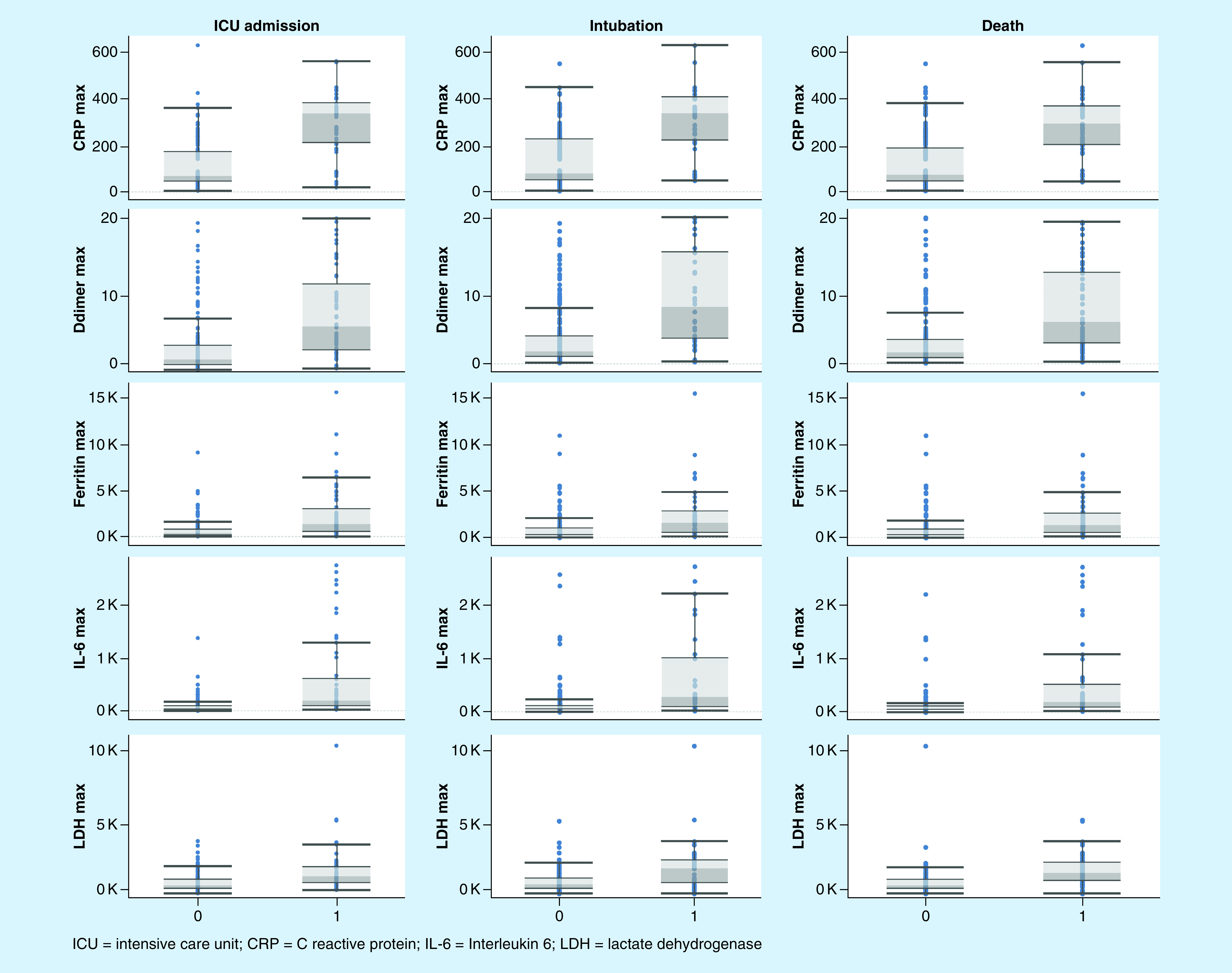
Whisker plots for different biomarkers and outcomes. ICU: Intensive care unit.

Univariate logistic regression models yielded statistically significant estimates for IL-6 ≥ 60 pg/ml, D-dimer ≥ 3 μg/ml, CRP ≥ 90 mg/l, ferritin ≥ 450 ng/ml and LDH ≥ 1200 units/l for Intensive Care Unit (ICU) admission, intubation and death. The odds ratio of the three outcomes with the different markers is illustrated in Table 3. Hypertension, heart disease and neurologic disorders had significant association with ICU admission, intubation and death. The other medical conditions were not significant, except for chronic kidney disease having a significant association with death. Male sex had a higher odds ratio of intubation and ICU transfer than female sex. A separate stratified analysis was conducted for every marker with respect to the comorbid conditions to eliminate any confounding effect. The Mantel–Haenszel test for heterogeneity was not significant except for cancer (hematologic and solid malignancies) with respect to D-dimer, and sex with respect to IL-6. The adjusted odds ratios are thus reported in Table 3. The median values for each of the outcomes are represented in Table 4 and the distribution of the data is illustrated in Figure 1.

**Table 3.** Odds ratios for all biomarkers with 95% confidence intervals.

Biomarker	OR for ICU admission	OR for intubation	OR for death
IL-6 ≥50 pg/ml (0.0–15.5 pg/ml)	5.9 (2.7, 13.1)^†^	4.6 (1.7, 12.4)^†^	5.4 (2.4, 11.8)^†^
D-dimer ≥3 μg/ml (0.20–0.28 μg/ml)	7.3 (3.8, 14.0)^†^	8.2 (3.4, 19.8)^†^	7.5 (3.8, 14.6)^†^
Ferritin ≥450 ng/ml (20–450 ng/ml)	6.8 (3.4, 13.7)^†^	4.0 (1.8, 8.8)^†^	5.1 (2.6, 10.0)^†^
CRP ≥100–mg/l (0.0–9.0 mg/l)	8.6 (4.1, 18.1)^†^	6.5 (2.6, 16.3)^†^	6.0 (3.0, 12.0)^†^
LDH ≥1200 (600–800 units/l)	4.5 (2.5, 8.1)^†^	5.4 (2.5, 11.8)^†^	8.0 (4.1, 15.3)^†^

^†^ indicates statistical significance at p-value of 0.05.

ICU: Intensive care unit; OR: Odds ratio.

**Table 4.** Median Biomarker for each outcome.

Biomarker median value	ICU admission	No ICU admission	Intubation	No intubation	Death	Survival
IL-6 pg/ml (0.0–15.5 pg/ml)	188	50	266	60	188	50
D-dimer μg/ml (0.20-0.28 μg/ml)	5.9	1.6	7.8	1.7	5.8	1.6
Ferritin (20-450 ng/ml)	1360	340	1575	373	1320	356
CRP mg/l (0.0-9.0 mg/l)	333	68	333.2	78	290	73
LDH (600-800 units/l)	1478	870	2050	915	1680	863

ICU: Intensive care unit.

## Discussion

Although population-based studies have identified risk factors for poor prognosis in COVID-19, the clinical course of individual patients infected with the virus is highly variable. Risk stratification of medical comorbidities as well as biologic markers predicting clinical deterioration are needed for the US population.

In our cohort of patients, the only comorbidities associated with poor outcomes across all three end points were history of hypertension, cerebrovascular disease and heart disease. Chronic kidney disease was associated with an increased odds ratio of death. Other analyses have demonstrated an association between obesity and diabetes as having poor outcomes in hospitalized patients with COVID-19 infection [5,6,11]. We suspect that our cohort of patients was too small to detect any other significant comorbidities.

Our retrospective study did identify an association between biomarkers and clinical deterioration as well as death. Elevated levels of IL-6, D-dimer, CRP, LDH and ferritin all had an independent increased risk for the clinical outcomes assessed (ICU admission, invasive ventilatory support and death), which were statistically significant. Specifically, in our cohort, these biomarkers remained statistically significant in patients with hypertension and cardiovascular disease. These associations were also independent of any patient comorbidities, except for cancer which appeared to have an effect on D-dimer. However, the prevalence of cancer in this population was low. Thus, a higher number of patients with this condition are required to confirm the confounding effect of cancer on D-dimer.

Of further significance is that our cohort of COVID-19 patients admitted to the hospitalist service revealed that the highest odds of death occurred when the LDH level was greater than 1200 units/l and a D-dimer level was greater than 3 μg/ml. Though more modest, all other biomarkers including IL-6, ferritin and CRP also carried a statistically significant increase in odds of death. These findings appear to corroborate that the inflammatory indices of IL-6, CRP and ferritin are independently associated with patients becoming critically ill, requiring ventilatory support and dying. It remains unclear whether these inflammatory indicators are biologic markers of disease or mediators of the hypothesized ‘cytokine storm,’ in which excessive cytokine release results in hyperinflammation and multi-organ disease [12]. However, there are no studies to aid in contextualizing the significance of inflammatory biomarkers. It is possible these markers predict clinical course and if so, they could inform therapeutic interventions rather than simply demonstrate a consequence of the disease. Further study on the timing and frequency of measuring inflammatory biomarkers, and the value of trending them as it relates to clinical outcomes, is clearly warranted.

In our cohort, the odds of intubation were the highest with a D-dimer greater than 3 μg/ml. These patients had a higher likelihood of clinically deteriorating and transferring to the ICU, requiring mechanical ventilatory support and greater odds of death. Our decision to use a D-dimer threshold of 3 μg/ml, rather than the median of 2.1 μg/ml, was based on a study pointing to a D-dimer threshold of 3 μg/ml as having a higher specificity for identifying patients with thrombotic complication [10].

The clinical predictive value of an elevated D-dimer corroborates findings observed in prior studies [6,13] where an elevated D-dimer was associated with an increased risk of needing ventilator support and in-hospital mortality. Limited histopathologic and autopsy data showed evidence of exudative inflammation, small vessel occlusion and a thrombotic microangiopathy, suggestive of a hypercoagulable state [14–16]. In addition, a single retrospective study has demonstrated a mortality benefit in patients with high D-dimer levels (six-times the upper limit of normal) who were treated with prophylactic doses of low molecular weight heparins [13]. Of note, the mortality rate in their patients was substantially higher than that observed in our cohort, suggesting that we may be already achieving a benefit from the deep venous thrombosis prophylaxis, that is routinely used in the US, in contrast to the clinical practice in China. The cohort of patients that Tang *et al.* reviewed were not routinely treated with chemoprophylaxis for prevention of deep venous thrombosis [13]. Whether therapeutic anticoagulation with heparin would yield greater benefit has yet to be demonstrated and deserves further clinical study.

Our study has some limitations. First, not all laboratory tests were done on all patients and, as a result, the role of an individual marker might be overestimated. This is largely due to the lack of a standardized clinical practice for COVID-19. Obtaining these biomarkers was not the standard of care at the time given the lack of supporting evidence for clinical utility. Thus, in the early days of the pandemic, these biomarkers were not measured in all patients. As the care of COVID-19 patients became standardized, more clinicians began checking biomarkers.

Selection bias could overestimate the utility of these biomarkers. Our cohort did not include COVID-19 patients who were evaluated in the emergency room and deemed to be clinically well enough to return home. If biomarkers in these discharged patients were also elevated, their predictive value would be diminished. However, checking inflammatory biomarkers is not the standard of care in the emergency department. Perhaps the clinical context (referred for hospitalization) enhances the benefit of these biomarkers for an inpatient team.

Furthermore, this study uses cutoff points for the evaluation of the different biomarkers, rather than their absolute value. This is largely due to our limited sample size, to weigh each biomarker appropriately. However, a simple cut-off point enhances the clinical applicability of this study by creating a straightforward way for clinicians to identify the sickest patients.

Despite these limitations, we believe these markers can aid clinicians in identifying hospitalized COVID-19 patients at risk of clinical deterioration. Our findings suggest that monitoring IL-6, D-dimer, CRP, LDH and ferritin has clinical utility in this respect, especially when these markers are above the cutoff values mentioned. Following these biomarkers could ensure closer monitoring of these patients and provide guidance and standardization in the allocation of increasingly scarce resources.

## Conclusion

Laboratory markers of inflammation and coagulopathy can help clinicians identify patients who are at a high risk for clinical deterioration. Future directions of research could focus on the temporal variability of these biomarkers and outcomes in COVID-19 patients, as well as, the effect of treatment with full dose anticoagulation.

**Summary points**

**Background**

- The global pandemic caused by novel coronavirus disease (COVID-19) remains poorly understood by clinicians.
- Identifying biologic markers associated with prognosis can help clinicians recognize disease severity.
- The clinical impact of these biological markers on outcomes in the US population remains unclear.

**Materials & methods**

- A retrospective cohort of COVID-19 patients were identified, their admission and peak biomarkers were extracted and the odds of adverse clinical outcomes were calculated.

**Results**

- A total of 299 patients were identified with a mortality of 24% and 200 patients had a full biomarker panel check.
- IL-6, CRP, ferritin, lactate dehydrogenase and D-dimer threshold levels all had an independent increased odds of intensive care unit transfer, intubation requirement and death.
- Lactate dehydrogenase had the highest odds associated with patient mortality, followed by D-dimer.
- CRP and D-dimer had the highest odds associated with intensive care unit transfer and intubation, respectively.

**Conclusion**

- Biomarkers of inflammation and coagulopathy can aid in identifying hospitalized COVID-19 patients at risk for clinical deterioration.
